# Public Trust in Artificial Intelligence Applications in Mental Health Care: Topic Modeling Analysis

**DOI:** 10.2196/38799

**Published:** 2022-12-02

**Authors:** Yi Shan, Meng Ji, Wenxiu Xie, Kam-Yiu Lam, Chi-Yin Chow

**Affiliations:** 1 Nantong University Nantong China; 2 University of Sydney Sydney Australia; 3 City University of Hong Kong Hong Kong China

**Keywords:** public trust, public opinion, AI application, artificial intelligence, mental health care, topic modeling, topic, theme, term, visualization, user feedback, user review, Google Play, health app: mHealth, mobile health, digital health, eHealth, mental health, mental illness, mental disorder

## Abstract

**Background:**

Mental disorders (MDs) impose heavy burdens on health care (HC) systems and affect a growing number of people worldwide. The use of mobile health (mHealth) apps empowered by artificial intelligence (AI) is increasingly being resorted to as a possible solution.

**Objective:**

This study adopted a topic modeling (TM) approach to investigate the public trust in AI apps in mental health care (MHC) by identifying the dominant topics and themes in user reviews of the 8 most relevant mental health (MH) apps with the largest numbers of reviewers.

**Methods:**

We searched Google Play for the top MH apps with the largest numbers of reviewers, from which we selected the most relevant apps. Subsequently, we extracted data from user reviews posted from January 1, 2020, to April 2, 2022. After cleaning the extracted data using the Python text processing tool spaCy, we ascertained the optimal number of topics, drawing on the coherence scores and used latent Dirichlet allocation (LDA) TM to generate the most salient topics and related terms. We then classified the ascertained topics into different theme categories by plotting them onto a 2D plane via multidimensional scaling using the pyLDAvis visualization tool. Finally, we analyzed these topics and themes qualitatively to better understand the status of public trust in AI apps in MHC.

**Results:**

From the top 20 MH apps with the largest numbers of reviewers retrieved, we chose the 8 (40%) most relevant apps: (1) Wysa: Anxiety Therapy Chatbot; (2) Youper Therapy; (3) MindDoc: Your Companion; (4) TalkLife for Anxiety, Depression & Stress; (5) 7 Cups: Online Therapy for Mental Health & Anxiety; (6) BetterHelp-Therapy; (7) Sanvello; and (8) InnerHour. These apps provided 14.2% (n=559), 11.0% (n=431), 13.7% (n=538), 8.8% (n=356), 14.1% (n=554), 11.9% (n=468), 9.2% (n=362), and 16.9% (n=663) of the collected 3931 reviews, respectively. The 4 dominant topics were topic 4 (cheering people up; n=1069, 27%), topic 3 (calming people down; n=1029, 26%), topic 2 (helping figure out the inner world; n=963, 25%), and topic 1 (being an alternative or complement to a therapist; n=870, 22%). Based on topic coherence and intertopic distance, topics 3 and 4 were combined into theme 3 (dispelling negative emotions), while topics 2 and 1 remained 2 separate themes: theme 2 (helping figure out the inner world) and theme 1 (being an alternative or complement to a therapist), respectively. These themes and topics, though involving some dissenting voices, reflected an overall high status of trust in AI apps.

**Conclusions:**

This is the first study to investigate the public trust in AI apps in MHC from the perspective of user reviews using the TM technique. The automatic text analysis and complementary manual interpretation of the collected data allowed us to discover the dominant topics hidden in a data set and categorize these topics into different themes to reveal an overall high degree of public trust. The dissenting voices from users, though only a few, can serve as indicators for health providers and app developers to jointly improve these apps, which will ultimately facilitate the treatment of prevalent MDs and alleviate the overburdened HC systems worldwide.

## Introduction

### Background

“Mental disorders are one of the greatest public health concerns of our time” [1]. It is estimated that mental disorders (MDs) account for 32.4% of years lived with disability and 13.0% of disability-adjusted life years [2]. MDs have a lifetime prevalence ranging from 12% to 47.4% worldwide [3]. They affect millions of people worldwide, imposing a heavy burden on health care (HC) systems [4]. The heavy burden of MDs calls for increasing mental health (MH) research worldwide [1].

A possible resolution to this burden is adopting mobile health (mHealth) technologies, especially mobile phone apps [4]. Mental health care (MHC) is “having a digital moment” [5]. Digital mental health care (DMHC) takes diverse forms, varying from online psychotherapy (eg, cognitive behavioral therapy) to chatbots, which are computer programs using artificial intelligence (AI) and natural language processing to engage in conversations with text or text to speech [6]. These AI apps in MHC boast unmatched advantages, including “increased convenience for patients, enhanced adherence to appointments, and access to care that is unbound by geography, backed by evidence that digital mental health care can be effective” [5]. Providing evidence-based care, AI health apps have become increasingly popular, evidenced by over 150,000 downloads of an app named PTSD Coach [7]. However, if not guided by human therapists, they have high dropout rates [8]. By investigating the most popular MH apps downloaded over 100,000 times, Baumel et al [9] discovered that 96% of users no longer engaged with the apps after 2 weeks. This is because DMHC may not guarantee “patient privacy, confidentiality, and reliability of service delivery” [5]. According to previous studies, what influences the implementation of AI apps includes (1) people-related factors (eg, public attitudes, trust), (2) health system–related factors (eg, clinical responsibility and accountability, the possibility of harm, and issues of regulation and service provision), (3) data-related factors (eg, issues of data security, privacy, consent, and ownership), and (4) tool-related factors (eg, issues of reliability and validity) [10-12].

“[The] COVID-19 pandemic has changed health care delivery, including mental health care services around the world” [5]. DMHC has been “a welcome and much-needed adoption” in the current pandemic, mainly because no safe alternative can provide MH services. DMHC helps to sustain core MH services. In this state of research, to what extent do users trust AI apps in MHC?

### Trust Defined and Trust in AI Systems (Apps)

As an important social lubricant for cooperative behavior [13], trust has been investigated in various disciplines, which have provided varying definitions, leading to “a multidimensional family of trust concepts,” each with a specific focus [13]. Trust is fundamentally “a feeling of certainty that a person or a thing will not fail,” often based on inconclusive evidence and categorized into interpersonal trust, social trust, and trust in automation [14]. It is 1 of the means that people can use to reduce complexity in a complex world by decreasing the number of choices to be considered in particular circumstances [15,16]. Since trust can reduce fear, risk, and complexity both online and offline, it is inconceivable that a robust, interactive online environment would be possible without trust [13].

The concept of human trust has been investigated in automated systems [17] and online systems [13]. Reeves and Nass [18] studied how people treated new technologies as humans and as objects of trust, finding that people responded to these technologies almost in the same way that they responded to real people in social relationships by behaving politely or rudely to computer systems; regarding them as assertive, timid, or helpful agents; and responding to them physically. Trust in technology is the belief that a tool, machine, or equipment will not fail [19]. “Trusting relationships with the technology have the potential to affect the way the technology is used or not used” [16]. The literature on trust in HC systems focused on patient-physician interpersonal trust [12,20,21] and patients’ trust in HC systems [22,23]. Most research on trust in technology pointed to the trust of the technology operators, for example, the care providers (physicians, nurses, or technicians) in medical settings [24]. However, trust in technology needs to be studied from the perspective of users. It is their trust in automated systems that results in appropriate use, disuse, misuse, or even abuse of the automation [25].

Trust in information sources plays an essential part in making the individual well informed of information and willing to act upon it [26]. For example, people perceive their risks of COVID-19 infection differently, depending on whether the information is received from social media or from mass media [27]. It has been found that messages from privately affiliated media sources can undermine people’s trust in scientific knowledge and health policies [28]. Trust is an essential factor that impacts human interactions with AI [29]. It is crucial to understand human-AI trust dynamics, especially in the HC domain, in which life may be at risk [29]. User perception of AI systems’ capabilities is always a significant factor for trust in them [29], and the degree of trust in them considerably influences the level of user reliance on them [30] and thus the user efficacy of HC decisions [29]. Reliability (ie, AI systems’ predictable and consistent performance of tasks [31]) is particularly concerning in HC because AI changes in reliability in the presence of new data [32]. The reliability of AI systems is also conditioned both by input data and by user data [29]. When trained with insufficient and subjective data from diverse sources, AI systems might generate overfitted or even biased outcomes of which clinical users could not be aware [29]. These factors undermine the performance of AI technology [33], preventing users from trusting and accepting AI systems [29]. Understanding trust in AI systems will shed light on decision-making concerning the acceptance or rejection of technologies, the system designs bringing about positive patient outcomes, and those imposing adverse effects [14].

To the best of our knowledge based on literature search and review, no study has exclusively investigated public trust in AI apps or systems in MHC, although some studies have examined trust in medical technology [14]; human trust in AI in HC [29]; public trust in COVID-19–related government information sources (eg, the US Centers for Disease Control and Prevention), private sources (eg, FOX and CNN), and social networks (eg, Facebook and Twitter) [34]; and public trust in COVID-19–tracing apps [35]. In this state of research, there is a pressing need to explore public trust in AI apps or systems in MHC, particularly in the current context where MDs are listed among the greatest public health concerns of our times [1].

### Objective

This study aims to investigate public trust in AI apps in MHC by identifying the major topics (themes) in users’ reviews of the 8 most relevant MH apps with the largest numbers of reviewers.

Based on this research objective, we proposed the following research questions:

- Research question 1: Did the users trust the 8 most reviewed MH apps?
- Research question 2: What dominant topics (themes) and most relevant terms could be identified in user reviews?
- Research question 3: Were the identified dominant topics (themes) concerned with public trust or mistrust?
- Research question 4: What implications can the identified trust or mistrust provide for developers and providers of MH apps?

Topic modeling (TM) was adopted to process the reviews of these apps. As a statistical model, TM arranges unstructured data structurally using latent themes [36]. With this model, we scrutinized the reviews of the apps under discussion to reveal the extent to which users trusted these apps. The findings could not only fill the gap in the literature but also facilitate the cooperation between HC practitioners and app developers to design MH intervention programs for these MH apps to respond to public concerns effectively.

## Methods

### Data Collection

We searched Google Play for the top 20 MH apps with the largest numbers of reviewers, from which we selected the 8 (40%) most relevant apps: (1) *Wysa: Anxiety Therapy Chatbot*; (2) *Youper Therapy*; (3) *MindDoc: Your Companion*; (4) *TalkLife for Anxiety, Depression & Stress*; (5) *7 Cups: Online Therapy for Mental Health & Anxiety*; (6) *BetterHelp-Therapy*; (7) *Sanvello*; and (8) *InnerHour*. Subsequently, we extracted the data of user reviews of these apps posted from January 1, 2020, to April 2, 2022, by using a Python web crawler. After extracting the data, we created a data set of 3931 reviews (reviews of less than 20 words were removed) that consisted of the names of the selected apps, the content of these reviews, the date when these reviews were published, the dominant topic in each review, the contribution of the dominant topic to each review measured in percentage points, and the terms related to each topic. The data set is given in Multimedia Appendix 1. Before the analysis, we followed the standard preprocessing procedures designed in previous studies [37,38] to clean the data using Python 3.0 (Python Software Foundation) and to perform word part-of-speech tagging and text processing using the Python library *spaCy* [39,40]. Through data cleaning, we converted the words in the reviews into lowercase words; removed stop words, punctuation, numbers, and nonword characters; and stemmed the remaining text [41]. To generate more interpretable topics of high quality, we restricted the parts of speech of words to “noun” (NOUN), “verb” (VERB), “adjective” (ADJ), or “proper noun” (PROPN). The standard preprocessing procedures can significantly enhance the performance of algorithms and stabilize the stochastic inference of latent Dirichlet allocation (LDA) [38].

### Topic Modeling With LDA

The statistical methods of unsupervised TM algorithms (which do not need prior labeling or annotations of the documents) were designed to analyze the words (terms) of the original texts to identify the themes (topics) running through a corpus [42,43]. These algorithms allow users to organize and summarize numerous documents that cannot be annotated manually [41], thereby revealing the hidden topics in the documents [43]. We adopted the LDA TM technique, which assumes that texts are generated from a mixture of topics [44]. LDA is efficient and can generate topics of better quality [45]. From the data set created, we generated 2 probability distribution outputs: the probability distribution of topics over documents and the probability distribution of terms over topics [41,43]. The number of topics was determined by repeating the analysis with different numbers of topics and by comparing the perplexity of each analysis [41]. A lower perplexity value indicates a better model fit [44], and the perplexity value decreases with the increase in the number of topics [41]. Both the simplicity and the interpretability of the textual content need to be considered in choosing the optimal number of topics [38].

**Figure 1:**
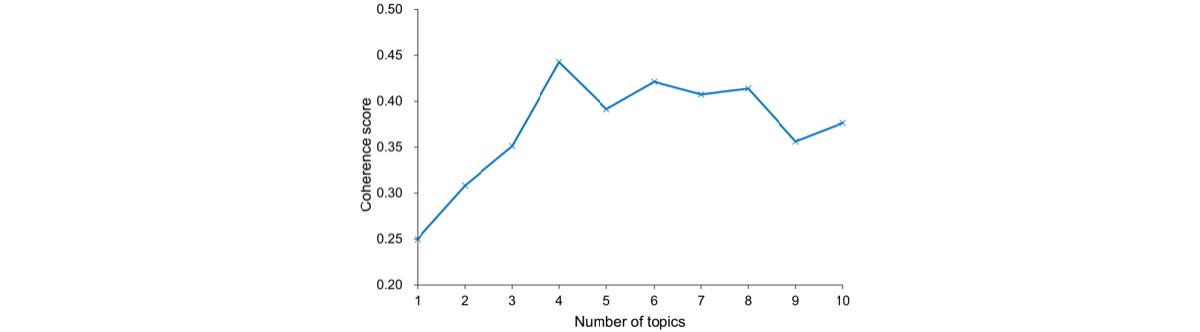
Coherence score for the topic numbers.

We took the coherence score as an assessment metric to evaluate how good a given topic model was and determine the optimal number of topics [46] that needed to be extracted from the user reviews. Topic coherence is a qualitative method used to score the coherence of a given topic [46]. It measures the semantic similarity between words with high scores in a topic to determine the consistency of a single topic, improving the semantic understanding of the topic [36]. We applied the Python package coherence model from *Gensim* to calculate the coherence value [47]. As shown in Figure 1, the coherence score increased to the highest value of 0.45 when the number of topics reached 4, before decreasing gradually, implying that the optimal number of topics was 4. Afterward, we visualized the relationship between these 4 topics and their related terms using Python version 3.6.1 and the *pyLDAvis* tool [44].

**Figure 2:**
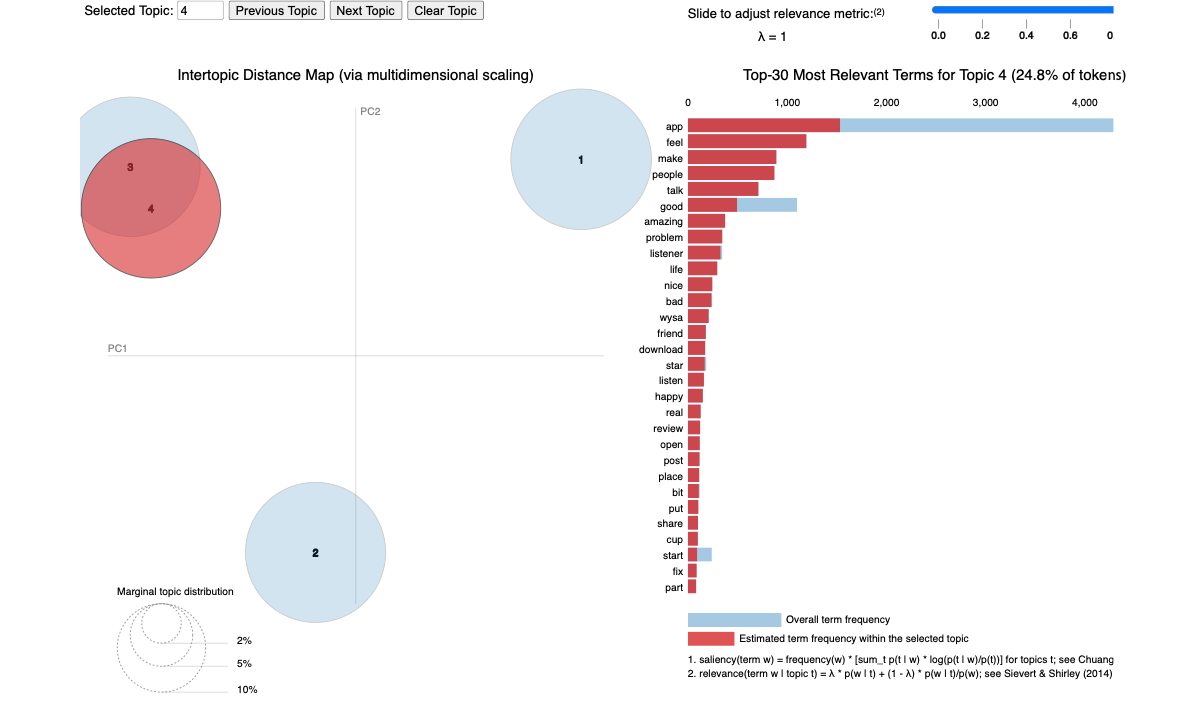
Intertopic distance map and top 30 most relevant terms for topic 4. Refer to the interactive web-based visualization in Multimedia Appendix 1 for other topics. PC: principal component.

When *λ* equals 1, terms are sorted according to their frequency in a topic. Therefore, we set *λ*=1 to visualize the intertopic distance between the 4 topics and the top 30 most relevant terms for each topic, as shown in Figure 2. We classified these 4 topics into different themes to facilitate better analysis based on the computed topic distance [44]. In the 2D plane (Figure 2), the 4 topics are shown in the form of 4 circles. The size of each circle represents the overall prevalence of the topic, the overlap between circles 3 and 4 means the overlap between topics 3 and 4, and the distance between the circle centers stands for topic distance [44]. The content of each topic was generated according to its corresponding set of keywords (terms) [48]. Considering that the output of statistical measures cannot be guaranteed to be interpretable due to the language complexity [49], we complemented automatic text statistics with manual interpretation when analyzing the topics. The topics were named based on the associated keywords to illustrate those topics [48].

### Ethical Considerations

All reviewers anonymized themselves when posting app reviews online. We just downloaded the anonymized data from the apps. Therefore, an ethical review was not necessary for this paper.

## Results

### Review Distribution Over Apps and Time

**Figure 3:**
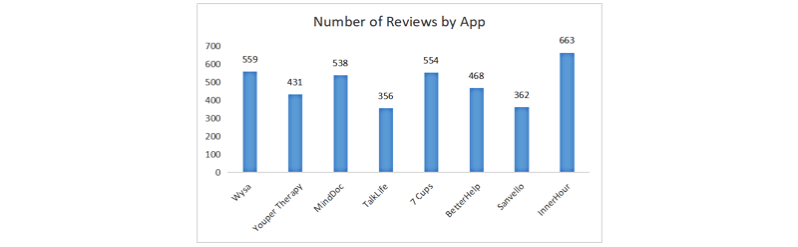
Number of reviews by app.

**Figure 4:**
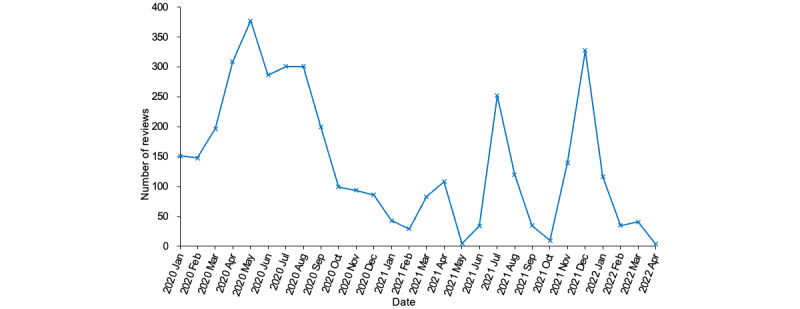
Number of reviews over time.

Figure 3 shows the distribution of reviews over the 8 selected apps. Over the period from January 1, 2020, to April 2, 2022, the largest number of reviews were posted on *InnerHour* (n=663, 16.9%), followed by *Wysa: Anxiety Therapy Chatbot* (n=559, 14.2%), *MindDoc: Your Companion* (n=538, 13.7%), and *7 Cups: Online Therapy for Mental Health & Anxiety* (n=554, 14.1%). Less than 450 reviews were published on *Youper Therapy* (n=431, 11%) and *BetterHelp-Therapy* (n=468, 11.9%). Far fewer than 400 reviews were released on *TalkLife for Anxiety* (n=356, 8.8%) and *Sanvello* (n=362, 9.2%). The different numbers of reviews somehow reflected the varying popularity of these 8 apps over the period under discussion. Figure 4 displays the number of reviews published over time, which peaked and waned on different dates. Although it is difficult to attribute the 2 peaks in 2021 to specific factors, it is likely that the peaking period in the middle of 2020 possibly reflected the increased need for MHC apps during the global outbreak of the COVID-19 pandemic, as evidenced by the following reviews:

It’s really helpful specially during this pandemic. You can find very calm listeners who will be patient and understanding while listening.

Love this app. Tried it because of a friend’s recommendation and best decision in this challenging time of a pandemic. Really cleared my mind and helped me regain control and life perspective. Will make it a daily habit.

This app is perfect for these times. With anxiety issues predating the pandemic, I'll probably continue using it after the world gets back on track. Thank you!

### Topic Prevalence

By applying LDA TM, we classified into 4 topics the collected data of user reviews of the 8 selected AI apps in MHC published from January 1, 2020, to April 2, 2022 (Table 1). Topic 4 (*cheering people up*) was the most popular and dominant topic, occurring in 27.2% (n=1069) of the 3931 reviews collected, closely followed by topic 3 (*calming people down*), which appeared in 26.2% (n=1029) of the 3931 reviews. Topic 2 (*helping figure out the inner world*) ranked as the third-most popular and dominant topic, showing up in 24.5% (n=963) of the 3931 reviews. Topic 1 (*being an alternative or complement to a therapist*) accounted for 22.1% (n=870) of the 3931 reviews. The similar areas of the 4 circles representing these 4 topics in Figure 2 indicate the similar overall prevalence of these topics in the collected reviews.

**Table 1:** Topic classification and keywords.

Theme and classification			Keywords	Reviews (N=3931), n (%)
**Theme 1: being an alternative or complement to a therapist**				
	Topic 1: being an alternative or complement to a therapist	great, therapist, time, work, therapy, issue, chat, counselor, easy, option, week, person, experience, money, support, month, session, message, year, service, start, worth, account, enjoy, video, conversation, update, access, call, log		870 (22.1)
**Theme 2: helping figure out the inner world**				
	Topic 2: helping figure out the inner world	helpful, day, give, find, good, app, mood, time, question, feeling, mental_health, track, thought, change, emotion, answer, phone, notification, check, add, daily, tool, positive, community, hard, journal, provide, insight, negative, advice		963 (24.5)
**Theme 3: dispelling negative emotions**				
	Topic 3: calming people down	app, love, thing, pay, lot, free, anxiety, recommend, depression, premium, feature, understand, stress, calm, AI^a^, version, hope, struggle, care, exercise, meditation, sleep, afford, activity, improve, good, offer, a lot, awesome, deal		1029 (26.2)
	Topic 4: cheering people up	app, feel, make, people, talk, good, amazing, problem, listener, life, nice, bad, Wysa, friend, download, star, listen, happy, real, review, open, post, place, bit, put, share, cup, start, fix, part		1069 (27.2)

^a^AI: artificial intelligence.

### Intertopic Distance

Figure 2 provides an overview of the topic model we constructed. The 4 circles in this figure represent the 4 topics dominating the 3931 reviews. The intertopic distances characterized by multidimensional scaling on the 2D plane in Figure 2 imply the semantic similarity between these 4 topics: topics 3 and 4 overlap and are thus semantically similar; these 2 topics are semantically distant from topics 1 and 2, which are also semantically distant from each other.

### Topic Terms and Content

Figure 2 also shows the top 30 most relevant terms for topic 4, representing 24.8% of the tokens. As this topic constitutes the highest proportion (n=1069, 27.2%) of the collected reviews, it is presented as a typical illustration. Since the blue bar and the red bar represent the overall term frequency and the estimated term frequency within the selected topic, respectively, the topic content can be better interpreted based on this approach [50,51]. With regard to topic 4, users of the 8 selected AI apps in MHC preferred to use words indicating the apps’ function of cheering people up, such as *make*, *feel*, *happy*, *good*, *amazing*, *listener*, *friend*, and *nice*. In this way, we could study the content of this topic and name it. The word clouds of the 4 dominant topics in Multimedia Appendix 2 complement Figure 2 in demonstrating the term frequency of the top 30 most relevant terms of each topic and implying the content of each topic.

### Topic Classification and Keywords

Drawing on TM to analyze the user reviews and compare the perplexity indices, we discovered that it was optimal to classify the 4 dominant topics covered in the reviews into 3 themes, as shown in Table 1. The illustrative quotes for these themes are displayed in Textbox 1.

Topic 1 (theme 1, “being an alternative or complement to a therapist”) deals with reviews that describe the apps’ function as an alternative or even a complement to a human MH provider (eg, nurse, doctor, physician, psychiatrist) when such a human therapist is unavailable due to the limitation of working time, space, budget, and social conditions (eg, the repeated resurgences of COVID-19); when app users attach great importance to privacy and social stigma related to MH problems; or when DMHC takes prevalence or will be institutionalized in the MHC domain. Topic 2 (theme 2, “helping figure out the inner world”) focuses on the apps’ role in helping users track their psychic world to gain a better understanding of their feelings and emotions. By expressing every emotional state to the apps, users can keep themselves in control emotionally, turning out to be a better, sounder self mentally. Topics 3 and 4 (theme 3, “dispelling negative emotions”) point to the emotion-soothing purpose of the apps under discussion. Specifically, these apps can serve to mitigate stress, anxiety, and depression that haunt users, effectively calming them down and cheering them up by behaving as a bosom friend, a good listener, and a constructive adviser, while avoiding judging them by what they say during their venting of various types of feelings.

**Illustrative quote(s) of each theme.**

**Theme 1**

- “Great! Very calming and I haven’t been able to see my therapist in a while and this really helped. Great quality too.”

**Theme 2**

- “This app has helped me a lot on my mental health. I feel much more in tune with myself and my emotions and all because this app helped me figure out myself and my inner world better. Plus I also have someone I can express everything at the end of the day.”

**Theme 3**

- “It’s a great help. It calms me down some and it’s calming to know that I wouldn’t be judged by what I say.”
- “Thanks to the Wysa team for making this! It’s been making it much easier for me to manage my stress (:”

## Discussion

### Principal Findings

MDs represent a tremendous public health concern of our times [1], which is increasingly overburdening the HC systems worldwide. This is especially true during the COVID-19 pandemic, which has caused many MDs worldwide [52]. In this context, Web 2.0–empowered DMHC apps are an optimal solution because there is currently a “tectonic shift in the ways in which patients consume health and medical information” [53]. Digital health interventions have proved effective in mitigating negative health habits and outcomes, leveraging patients’ growing proactivity in obtaining health information online [54]. This study is the first investigation of public trust in AI apps in MHC from the perspective of user reviews of MHC apps. Applying TM, we managed to manipulate unstructured user reviews, discovering 4 dominant topics hidden in a data set of 3931 reviews and categorizing these topics into 3 themes based on topic coherence and intertopic distance (semantic similarity).

The top 30 most relevant terms for the 4 dominant topics (Figures 2 and Multimedia Appendix 1) indicate that users of the 8 selected apps generally trust these apps, having a positive experience with these apps and feeling satisfied with them. Specifically, this was evidenced by the top terms *great*, *therapist*, *work*, *chat*, *counselor*, *easy*, and *option* in topic 1; *helpful*, *find*, *give*, *mood*, *good*, *question*, *positive*, and *feeling* in topic 2; *app*, *love*, *free*, *pay*, *lot*, *recommend*, *anxiety*, *depression*, and *stress* in topic 3; and *app*, *make*, *feel*, *people*, *talk*, *good*, *life*, *amazing*, *listener*, *nice*, *friend*, *open*, and *happy* in topic 4. In medical settings, trust results in enhanced health self-efficacy, increased treatment adherence, and more positive health outcomes [55,56]. This result confirms a previous research finding that overall user trust in conversational agents (CAs) is increasing [57].

The 4 topics that the users focused on were classified into 3 themes based on LDA TM. Theme 3 (*dispelling negative emotions*) covered topics 3 (*calming people down*) and 4 (*cheering people up*), which together dominated more than half (n=2098, 53.4%) of the reviews (N=3931) under scrutiny. This theme highlighted the apps’ perceived function of helping people out of depression, anxiety, and stress. The results revealed that the users are mainly concerned with positive MH outcomes when adopting AI apps for MH interventions. They sought social support messages concerning MH on MHC apps mainly because they trusted the apps in terms of (1) the look of the technology, (2) perceived reliability, (3) accuracy, (4) consistency, and (5) feedback from the technology [14], as demonstrated by the following reviews:

The app is also easy to navigate and seems solid (no tech issues). Also, nice professional appearance.

Very cute and user-friendly layout. Takes you step by step to teach you by example how to take the reins on your own thoughts and emotions. Awesome app!

Reliable. It’s a really great app, the listeners out on the platform are professional and patient. I have personally benefited from this app and I can't stress this enough, it is a really good app. If you just need an ear to listen to you, without any judgments whatsoever, try it.

It’s scary how meticulously accurate this personality test is. It is like having a paid friend Psychologist on hand during these uncertain times of total lockdown...

7 cups has consistently and diligently kept the essence of being there for anyone anywhere anytime, very alive and accessible

I love being able to message my counselor whenever I need to, it helps with needing to vent and get positive, constructive feedback quickly, on everyday stressors that feel overwhelming.

These quoted reviews show that some reviewers tend to trust computer systems more than other humans (ie, human MHC providers) because automation is expected to be relatively perfect [58], in terms of appearance, reliability, accuracy, consistence, and targeted feedback [14].

Moreover, our results confirmed that *helping figure out the inner world* (theme 2) was another important factor determining public trust. Reviewers repeatedly mentioned *thought tracking*, *best mood tracker*, *journalizing my thoughts and feelings*, *making me take a proper look at my thoughts and feelings*, *writing down my thoughts and then framing them to be more positive*, etc. The function of enabling users to follow through with their MH made them trust their MH with these apps due to the negative social perception of MDs [59], which prevents people from seeking professional help proactively [60]. This result aligns with previous studies [4,59] that mHealth technologies (eg, MH apps) can protect users’ privacy and reduce the influence of the social stigma attached to MDs. Users commented that “it can be nice to vent to something that isn’t someone who would judge you.” In addition, people are most likely to depend on the advice and support provided by professional apps when developing appropriate responses to MH problems [54]. The professional interventions in thought tracking supplied by the selected apps were another essential contributor to the high degree of public trust in these apps, as evidenced by some reviews, such as:

The bot navigates you through series of practical and professional activities

Very professional

Professional help

Helps me assemble my thoughts by asking me relevant questions

Additionally, our research results suggest that MH app users also reflect on the role of MH apps as an alternative or a supplement to human therapists (theme 1). This implies that app users attach great importance to digital health interventions, while still trusting human MHC providers. Although some users accepted MH apps as an alternative to human therapists, for example,

This app is my perfect personal therapist,

most users took them as a complement to human therapists, for example,

Very helpful between therapy appointments and during times when I wasn’t seeing a therapist.

The tectonic change in patients’ consuming health and medical information [53] significantly shifted the landscape for interventions seeking to change health behavior, each with its associated benefits and risks [61]. “Although exposure to these products is becoming ubiquitous, electronic health information is novel, incompletely disseminated, and frequently inaccurate, which decreases public trust” [54]. That is why a few users mistrusted the selected AI apps in their reviews:

It’s the same vague questions every time with no room for explanation. Very inaccurate

But the therapists seem very disconnected and don't really seem to care much at all, responding in broken or incomplete sentences

However, AI apps in MHC are gaining increasing popularity due to their unmatched advantages [5], the provision of evidence-based care [7], and the change in HC delivery due to the COVID-19 pandemic [5]. As a result, AI apps in MHC have become “a welcome and much-needed adoption” [5].

Admittedly, there were some dissenting voices in the collected reviews. These disagreements reflected some aspects of the selected apps that undermined public trust, including:

- Interface design

The interface sucks

- Charges

It was an exorbitant in-app purchase

- Engagement

...chat it was not engaged at all

- Reliability

Broken app. Chats are so unstable I couldn't get more than 2 messages before there was an error. The interface is broken, half of the stuff barely works and everything lags. The idea behind it is amazing but in practice it unfortunately doesn’t work at all.

- Ease of use

The app itself isn’t as easy to navigate compared to the desktop site

These negative comments of reviewers indicate that the reliability of service [5,29,31], AI systems’ capabilities [29], people-related factors (eg, public attitudes, trust), health system–related factors (eg, issues of regulation and service provision), and tool-related factors (eg, issues of reliability) [10-12] all potentially reduce public trust in DMHC delivered through AI apps or systems. Among these factors damaging public trust, reliability or predictable and consistent performance arouses particular concern in HC [32]. These findings are consistent with those reported in previous studies [5,10-12,29,31,32].

All these aspects need to be improved to enhance public trust. The technology design and the integration of technology into effectively performing systems are most likely to be associated with patient trust, which impacts patient outcomes (eg, patient satisfaction and adherence to medical advice and treatment) [62].

Overall, the investigation of user reviews based on the TM approach can shed light on public trust in AI apps in MHC. “Understanding trust in medical technologies will provide insight into decision making about which technologies will be accepted or rejected, which work system designs will lead to positive patient outcomes, and which will have the inverse effect” [14].

### Implications

Trust is an emotional construct that features patients’ comfortable feeling of faith in or dependence on care providers’ intentions, with common dimensions, including “competence, compassion, privacy and confidentiality, reliability and dependability, and communication” [63]. In the current and future context of technology replacing human elements in medical practices [14] and HC providers depending more on AI [29], a proper trust relationship [29] or calibrated trust [64] needs to be established between users and AI for HC for effective medical and health decisions [29]. To this end, important issues relevant to trust in AI for HC apps should be studied, including essential factors impacting trust in AI for HC, potential ways to improve trust relationships, and their influence on trust [29].

Currently, the adoption of AI systems in the HC domain is considerably hindered by a lack of trust in this technology [29]. Trust in AI is conditioned both by human factors and by properties of AI systems. Human factors include users’ education, experiences, personal biases, and perception of automation [29]. Properties of AI systems involve the look of the technology, perceived reliability, accuracy, consistency, and feedback from the technology [14]. Considering these contributors to public trust in AI systems, we propose that patients be provided with education and information about the technology and its use [14] and that developers of AI apps for HC well consider health system–related factors, data-related factors, and tool-related factors [10-12] when designing HC apps and systems. In addition, mechanisms need to be incorporated into the development of AI to build and keep an appropriately balanced, optimal level of user trust matching the capacities of AI systems [30].

### Limitations

This study may have some limitations. Most importantly, user reviews of the selected apps were subjective user comments based on the individual experience with the apps. Public trust in AI apps in MHC reflected by such self-reported data may be influenced by various factors, such as prior experience with the source, sociodemographic background, or health behaviors [54]. Thus, the user reviews may be biased, possibly influencing our research findings to some extent. Second, the user experience and satisfaction with AI apps in MHC are likely to be impacted by the current social situations. Therefore, public trust in such apps may vary according to changing social conditions, such as the current resurgences of the COVID-19 pandemic. Such changing status of public trust may thus not necessarily be triggered by the improved or deteriorating performance of the AI apps in MHC. The overall high degree of public trust found in this study may attenuate when the pandemic ends. Future studies need to consider the dynamics of the review data. Third, this study only retrieved reviews posted from January 1, 2020, to April 2, 2022. Further studies may expand this period to test the generalizability of our findings. Fourth, future research needs to consider more apps to verify the generalizability of the results of this study.

### Conclusion

This is the first study investigating the public trust in AI apps in MHC from the perspective of user reviews using the TM technique. The automatic text analysis and complementary manual interpretation of the collected reviews allowed us to discover 4 dominant topics hidden in a data set of 3931 unstructured reviews and categorize these topics into 3 groups of themes based on topic coherence and intertopic distance (semantic similarity). From these topics and themes, we managed to study the status of public trust in AI apps in MHC, finding an overall high degree of public trust. Although shaped by various factors, public trust in technology can broadly reflect the performance of the technology itself. The negative voices from users can serve as indicators for health providers and app developers to jointly improve these apps, which will ultimately facilitate the treatment of prevalent MDs and alleviate the overburdened HC systems worldwide. More research needs to be conducted into the design of AI apps for health counseling that ensures the validity of any provided recommendations and interventions. Meanwhile, patients must be reminded that health recommendations from any nonauthoritative sources need to be confirmed with HC professionals before they are acted on [65].

## Appendix

Multimedia Appendix 1 App review data and topic modeling results.

Multimedia Appendix 2 Topic Modeling word clouds of the 4 dominant topics.

## Abbreviations

AI: artificial intelligence
DMHC: digital mental health care
HC: health care
LDA: latent Dirichlet allocation
MD: mental disorder
MH: mental health
MHC: mental health care
mHealth: mobile health
TM: topic modeling

## References

[1] Motrico E, Salinas-Perez JA, Rodero-Cosano ML, Conejo-Cerón S (2021). Editors' comments on the special issue "Social Determinants of Mental Health". Int J Environ Res Public Health.

[2] Vigo D, Thornicroft G, Atun R (2016). Estimating the true global burden of mental illness. Lancet Psychiatry.

[3] Kessler RC, Angermeyer M, Anthony JC, DE Graaf R, Demyttenaere K, Gasquet I, DE Girolamo G, Gluzman S, Gureje O, Haro JM, Kawakami N, Karam A, Levinson D, Medina Mora ME, Oakley Browne MA, Posada-Villa J, Stein DJ, Adley Tsang CH, Aguilar-Gaxiola S, Alonso J, Lee S, Heeringa S, Pennell B, Berglund P, Gruber MJ, Petukhova M, Chatterji S, Ustün TB (2007). Lifetime prevalence and age-of-onset distributions of mental disorders in the World Health Organization's World Mental Health Survey Initiative. World Psychiatry.

[4] Shang J, Wei S, Jin J, Zhang P (2019). Mental health apps in China: analysis and quality assessment. JMIR Mhealth Uhealth.

[5] Gratzer D, Torous J, Lam RW, Patten SB, Kutcher S, Chan S, Vigo D, Pajer K, Yatham LN (2021). Our digital moment: innovations and opportunities in digital mental health care. Can J Psychiatry.

[6] Vaidyam AN, Wisniewski H, Halamka JD, Kashavan MS, Torous JB (2019). Chatbots and conversational agents in mental health: a review of the psychiatric landscape. Can J Psychiatry.

[7] Owen JE, Jaworski BK, Kuhn E, Makin-Byrd KN, Ramsey KM, Hoffman JE (2015). mHealth in the wild: using novel data to examine the reach, use, and impact of PTSD Coach. JMIR Ment Health.

[8] Gratzer D, Khalid-Khan F (2016). Internet-delivered cognitive behavioural therapy in the treatment of psychiatric illness. CMAJ.

[9] Baumel A, Muench F, Edan S, Kane JM (2019). Objective user engagement with mental health apps: systematic search and panel-based usage analysis. J Med Internet Res.

[10] Shaw J, Rudzicz F, Jamieson T, Goldfarb A (2019). Artificial intelligence and the implementation challenge. J Med Internet Res.

[11] Cresswell KM, Bates DW, Sheikh A (2013). Ten key considerations for the successful implementation and adoption of large-scale health information technology. J Am Med Inform Assoc.

[12] Greenhalgh T, Robert G, Macfarlane F, Bate P, Kyriakidou O (2004). Diffusion of innovations in service organizations: systematic review and recommendations. Milbank Q.

[13] Corritore CL, Kracher B, Wiedenbeck S (2003). On-line trust: concepts, evolving themes, a model. Int J Hum-Comput Stud.

[14] Montague EN, Winchester WW, Kleiner BM (2010). Trust in medical technology by patients and health care providers in obstetric work systems. Behav Inf Technol.

[15] Barber B (1983). The Logic and Limits of Trust.

[16] Lewis JD, Weigert A (1985). Trust as a social reality. Soc Forces.

[17] Madhavan P, Wiegmann DA, Lacson FC (2006). Automation failures on tasks easily performed by operators undermine trust in automated aids. Hum Factors.

[18] (1997). Computers & Mathematics with Applications.

[19] Sheridan TB (2002). Humans and Automation.

[20] Tarn DM, Meredith LS, Kagawa-Singer M, Matsumura S, Bito S, Oye RK, Liu H, Kahn KL, Fukuhara S, Wenger NS (2005). Trust in one's physician: the role of ethnic match, autonomy, acculturation, and religiosity among Japanese and Japanese Americans. Ann Fam Med.

[21] Arora NK, Gustafson DH (2009). Perceived helpfulness of physicians' communication behavior and breast cancer patients' level of trust over time. J Gen Intern Med.

[22] Zheng B, Hall MA, Dugan E, Kidd KE, Levine D (2002). Development of a scale to measure patients' trust in health insurers. Health Serv Res.

[23] Balkrishnan R, Hall MA, Blackwelder S, Bradley D (2004). Trust in insurers and access to physicians: associated enrollee behaviors and changes over time. Health Serv Res.

[24] Parasuraman R, Miller CA (2004). Trust and etiquette in high-criticality automated systems. Commun ACM.

[25] Parasuraman R, Riley V (2016). Humans and automation: use, misuse, disuse, abuse. Hum Factors.

[26] Flowers P, Riddell J, Boydell N, Teal G, Coia N, McDaid L (2019). What are mass media interventions made of? Exploring the active content of interventions designed to increase HIV testing in gay men within a systematic review. Br J Health Psychol.

[27] Lin Y, Hu Z, Alias H, Wong LP (2020). Influence of mass and social media on psychobehavioral responses among medical students during the downward trend of COVID-19 in Fujian, China: cross-sectional study. J Med Internet Res.

[28] Simonov A, Sacher S, Dubé J, Biswas S (2022). Frontiers: the persuasive effect of Fox News: non-compliance with social distancing during the COVID-19 pandemic. Mark Sci.

[29] Asan O, Bayrak AE, Choudhury A (2020). Artificial intelligence and human trust in healthcare: focus on clinicians. J Med Internet Res.

[30] Lee JD, See KA (2004). Trust in automation: designing for appropriate reliance. Hum Factors.

[31] Mcknight DH, Carter M, Thatcher JB, Clay PF (2011). Trust in a specific technology. ACM Trans Manage Inf Syst.

[32] Parikh RB, Obermeyer Z, Navathe AS (2019). Regulation of predictive analytics in medicine. Science.

[33] AlHogail A (2018). Improving IoT technology adoption through improving consumer trust. Technologies.

[34] Fridman I, Lucas N, Henke D, Zigler CK (2020). Association between public knowledge about COVID-19, trust in information sources, and adherence to social distancing: cross-sectional survey. JMIR Public Health Surveill.

[35] Oldeweme A, Märtins Julian, Westmattelmann D, Schewe G (2021). The role of transparency, trust, and social influence on uncertainty reduction in times of pandemics: empirical study on the adoption of COVID-19 tracing apps. J Med Internet Res.

[36] Liu Q, Zheng Z, Zheng J, Chen Q, Liu G, Chen S, Chu B, Zhu H, Akinwunmi B, Huang J, Zhang CJP, Ming W (2020). Health communication through news media during the early stage of the COVID-19 outbreak in China: digital topic modeling approach. J Med Internet Res.

[37] Lee TY, Bradlow ET (2011). Automated marketing research using online customer reviews. J Mark Res.

[38] Mankad S, Han HS, Goh J, Gavirneni S (2016). Understanding online hotel reviews through automated text analysis. Serv Sci.

[39] Day MY, Lee CC (2016). Deep learning for financial sentiment analysis on finance news providers.

[40] Zhao W, Luo X, Qui T (2018). Recent Developments in Smart Healthcare.

[41] Chen L, Wang P, Ma X, Wang X (2021). Cancer communication and user engagement on Chinese social media: content analysis and topic modeling study. J Med Internet Res.

[42] Blei DM (2012). Probabilistic topic models. Commun ACM.

[43] Liu L, Tang L, Dong W, Yao S, Zhou W (2016). An overview of topic modeling and its current applications in bioinformatics. Springerplus.

[44] Blei DM, Ng AY, Jordan MI (2000). Latent dirichlet allocation. J Mach Learn Res.

[45] Bogoradnikova D, Makhnytkina O, Matveev A, Zakharova A, Akulov A (2021). Multilingual sentiment analysis and toxicity detection for text messages.

[46] Stevens K, Kegelmeyer P, Andrzejewski D, Buttler D (2012). Exploring topic coherence over many models and many topics.

[47] GENSIM Topic Coherence Pipeline.

[48] Liu Q, Zheng Z, Chen J, Tsang W, Jin S, Zhang Y, Akinwunmi B, Zhang CJ, Ming W (2021). Health communication about hospice care in Chinese media: digital topic modeling study. JMIR Public Health Surveill.

[49] Grimmer J, Stewart BM (2017). Text as data: the promise and pitfalls of automatic content analysis methods for political texts. Polit Anal.

[50] Chuang J, Manning C, Heer J (2012). Termite: visualization techniques for assessing textual topic models. https://dl.acm.org/doi/abs/10.1145/2254556.2254572.

[51] Sievert C, Shirley K (2014). LDAvis: a method for visualizing and interpreting topics.

[52] Ransing R, Nagendrappa S, Patil A, Shoib S, Sarkar D (2020). Potential role of artificial intelligence to address the COVID-19 outbreak-related mental health issues in India. Psychiatry Res.

[53] Hesse BW, Nelson DE, Kreps GL, Croyle RT, Arora NK, Rimer BK, Viswanath K (2005). Trust and sources of health information: the impact of the internet and its implications for health care providers: findings from the first Health Information National Trends Survey. Arch Intern Med.

[54] Brown-Johnson CG, Boeckman LM, White AH, Burbank AD, Paulson S, Beebe LA (2018). Trust in health information sources: survey analysis of variation by sociodemographic and tobacco use status in Oklahoma. JMIR Public Health Surveill.

[55] Sbaffi L, Rowley J (2017). Trust and credibility in web-based health information: a review and agenda for future research. J Med Internet Res.

[56] Lee Y, Lin JL (2009). The effects of trust in physician on self-efficacy, adherence and diabetes outcomes. Soc Sci Med.

[57] Harrison C OK Google, Siri, Alexa, Cortana; Can you tell me some stats on voice search?.

[58] Seeger A, Heinzl A, Davis F, Riedl R, vom Brocke J, Leger P-M, Randolph A (2018). Human versus machine: contingency factors of anthropomorphism as a trustinducing design strategy for conversational agents. In F Davis, R Riedl, J vom Brocke, P-M Leger, A Randolph (Eds. Information Systems and Neuroscience.

[59] Angermeyer MC, Matschinger H (2003). The stigma of mental illness: effects of labelling on public attitudes towards people with mental disorder. Acta Psychiatr Scand.

[60] Ran M, Zhang T, Wong IY, Yang X, Liu C, Liu B, Luo W, Kuang W, Thornicroft G, Chan CL, CMHP Study Group (2018). Internalized stigma in people with severe mental illness in rural China. Int J Soc Psychiatry.

[61] Coulter A, Ellins J (2007). Effectiveness of strategies for informing, educating, and involving patients. BMJ.

[62] Safran DG, Kosinski M, Tarlov AR, Rogers WH, Taira DA, Lieberman N, Ware JE (1998). The primary care assessment survey: tests of data quality and measurement performance. Med Care.

[63] Pearson SD, Raeke LH (2000). Patients' trust in physicians: many theories, few measures, and little data. J Gen Intern Med.

[64] Hoffman RR, Johnson M, Bradshaw JM, Underbrink A (2013). Trust in automation. IEEE Intell Syst.

[65] Bickmore TW, Trinh H, Olafsson S, O'Leary TK, Asadi R, Rickles NM, Cruz R (2018). Patient and consumer safety risks when using conversational assistants for medical information: an observational study of Siri, Alexa, and Google Assistant. J Med Internet Res.

