# Supplementary figures and images for: Public Trust in Artificial Intelligence Applications in Mental Health Care: Topic Modeling Analysis
Source: JMIR Hum Factors. 2022 Dec 2;9(4):e38799. doi: 10.2196/38799 (PMC9758643; doi:10.2196/38799)

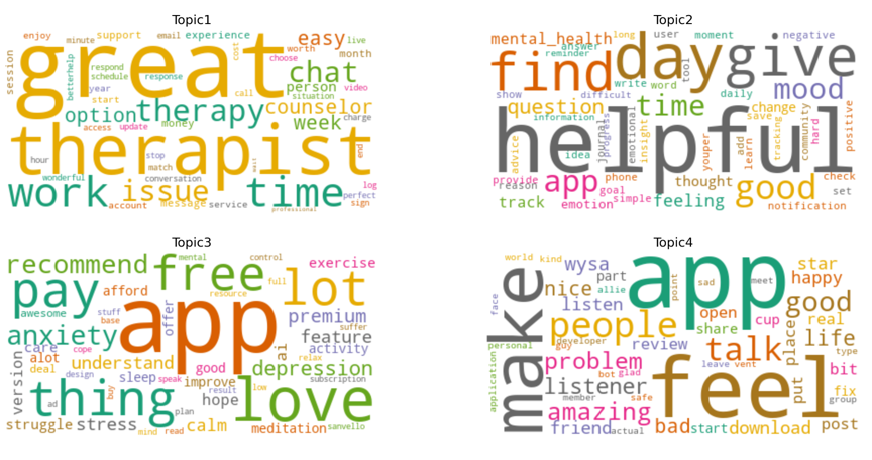

Supplement: Multimedia Appendix 2 [file humanfactors_v9i4e38799_app2.png]
